# Practical methods for improving dose distributions in Monte Carlo‐based IMRT planning of lung wall‐seated tumors treated with SBRT

**DOI:** 10.1120/jacmp.v13i6.4007

**Published:** 2012-11-08

**Authors:** Michael B. Altman, Jian‐Yue Jin, Sangroh Kim, Ning Wen, Dezhi Liu, M. Salim Siddiqui, Munther I. Ajlouni, Benjamin Movsas, Indrin J. Chetty

**Affiliations:** ^1^ Department of Radiation Oncology Henry Ford Health System Detroit MI USA

**Keywords:** SBRT, treatment planning, Monte Carlo, IMRT, lung

## Abstract

Current commercially available planning systems with Monte Carlo (MC)‐based final dose calculation in IMRT planning employ pencil‐beam (PB) algorithms in the optimization process. Consequently, dose coverage for SBRT lung plans can feature cold‐spots at the interface between lung and tumor tissue. For lung wall (LW)‐seated tumors, there can also be hot spots within nearby normal organs (example: ribs). This study evaluated two different practical approaches to limiting cold spots within the target and reducing high doses to surrounding normal organs in MC‐based IMRT planning of LW‐seated tumors. First, “iterative reoptimization”, where the MC calculation (with PB‐based optimization) is initially performed. The resultant cold spot is then contoured and used as a simultaneous boost volume. The MC‐based dose is then recomputed. The second technique uses noncoplanar beam angles with limited path through lung tissue. Both techniques were evaluated against a conventional coplanar beam approach with a single MC calculation. In all techniques the prescription dose was normalized to cover 95% of the PTV. Fifteen SBRT lung cases with LW‐seated tumors were planned. The results from iterative reoptimization showed that conformity index (CI) and/or PTV dose uniformity ($U_{\text{PTV}}$) improved in 12/15 plans. Average improvement was 13%, and 24%, respectively. Nonimproved plans had PTVs near the skin, trachea, and/or very small lung involvement. The maximum dose to 1cc volume (D1cc) of surrounding OARs decreased in 14/15 plans (average 10%). Using noncoplanar beams showed an average improvement of 7% in 10/15 cases and 11% in 5/15 cases for CI and $U_{\text{PTV}}$, respectively. The D1cc was reduced by an average of 6% in 10/15 cases to surrounding OARs. Choice of treatment planning technique did not statistically significantly change lung V5. The results showed that the proposed practical approaches enhance dose conformity in MC‐based IMRT planning of lung tumors treated with SBRT, improving target dose coverage and potentially reducing toxicities to surrounding normal organs.

PACS numbers: 87.55.de, 87.55.kh

## I. INTRODUCTION

Stereotactic body radiation therapy (SBRT), with its small targeted fields and high doses per fraction, has become an increasingly utilized modality in the treatment of medically inoperable lung cancer,^1^, ^4^ although several challenges still exist. Some of these issues lie in the underlying pathology of lung cancer itself. In lung cancer, high‐density tumor tissue is adjacent to surrounding lower‐density lung tissue. When irradiated, this dramatic dissimilarity in tissue density results in a loss of charged particle equilibrium (CPE) at the tumor/lung interface. This, in turn, yields a buildup of dose within the higher‐density tumor tissue on the radiation beam entrance side of the tumor and/or a builddown of dose on the beam exit side of the tumor.^5^, ^7^ Dosimetrically, the tumor tissue near the tumor/lung interface becomes underdosed relative to the portions of the tumor which are more interior to this buildup or builddown region. The distribution of this underdosed region depends on the location of the tumor relative to nearby structures.

**Figure 1:**
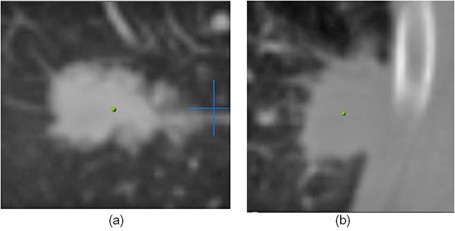
A CT slice image (a) of an island‐type lung tumor, which features the high‐density tumor completely surrounded by lower‐density lung parenchyma. A CT slice image (b) of a lung wall‐seated tumor in which the tumor abuts the chest wall and resides near lung‐adjacent OARs, such as the rib in this image.

**Figure 2:**
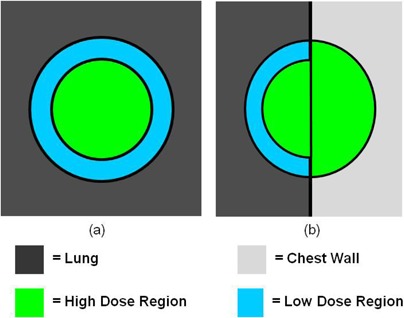
Schematic of high‐dose and low‐dose regions for a) island‐style lung tumors and b) lung wall (LW) seated tumors. When irradiated, a loss of CPE at the low‐density lung/high‐density tumor interface leads to a reduction of dose to the tumor around the lung‐embedded periphery. In the iterative reoptimization planning technique, the cold dose region for LW‐seated tumors is contoured and used as a boost structure.

Lung tumors treated by SBRT can be generally categorized in two groups: “island” type tumors ((Fig. 1a) which are completely surrounded by lung parenchyma, and lung wall (LW)‐seated tumors ((Fig. 1b), in which one side of the tumor is adjacent to the interface between the lung and the surrounding anatomical structures. When irradiated, island‐type tumors will feature a lower‐dose shell (or ring, in a single CT slice) surrounding a higher‐dose core ((Fig. 2a). In contrast, LW‐seated tumors will feature a partial low‐dose shell or ring along the lung embedded portion of the tumor and a more isotropic dose distribution towards the lung wall side which abuts more similar density tissues ((Fig. 2b). In the SBRT lung treatment planning of either case, it is of pivotal importance to ensure that the tumor/lung interface region is not underdosed.

A major limitation to this goal lies in the current state of commercially available treatment planning systems. Previously, most commercially available treatment planning systems used pencil beam (PB)‐based treatment planning algorithms for both their intensity‐modulated radiation therapy (IMRT) optimization and final dose calculation. However, the insufficiency of pencil beam (PB)‐based treatment planning algorithms has been widely noted in the literature, especially for interfaces between low‐ and high‐density tissues (such as between the lung and tumor) where they do not adequately account for electron transport and for conditions under which there is a loss of charged particle equilibrium (CPE).^8^, ^11^ In such situations, this results in incorrect dose calculations and has led many, including the AAPM, to recommend against using PB algorithms for SBRT lung dose calculations.^(9)^

Instead, the recommendation is to use treatment planning systems which employ alternative dose calculation algorithms, among the most accurate of which are Monte Carlo (MC)‐based.^10^, ^12^, ^13^ These algorithms use underlying physics principles to statistically assess the radiation beam/patient interaction and derive dose distributions which more accurately reflect reality compared to other algorithms, especially in situations with high‐density/low‐density interfaces, such as in the lung.^10^, ^12^, ^15^ MC‐based algorithms are becoming more widely available in commercial treatment planning systems, an example of which is the BrainLab iPlan treatment planning system.^11^, ^12^, ^15^, ^16^ However, given the current state of technology and computing power, there exists practical limitations to the clinical implementation of MC‐based algorithms. Due to their high computational expense, MC‐based dose calculations can be time‐consuming, causing issues for clinical workflow due to the large amount of computing power and personnel time required to create quality MC‐calculated treatment plans.^17^, ^18^

To make the applications more practical for everyday clinical use, current MC‐based treatment planning systems still use PB‐based algorithms when performing beamlet calculations in IMRT optimization, using MC only for the final dose calculation.^11^, ^19^, ^20^ Thus, such treatment plans may not be truly “optimized” in situations where PB works poorly, such as the lung, as the optimizer is making decisions based off inaccurate, PB‐derived dose distributions. As a result, goals set for the optimization process, such as ensuring that a percentage of the planning target volume (PTV) receives the prescription dose, may not be met. For IMRT‐based lung SBRT cases, the original planning goals attempting to get the prescription dose to the edges of the tumor along the lung/tumor interface are not met in the final MC dose calculation. The result is plans with the cold outer shell or semishell described above for island and LW‐seated tumors, respectively (Fig. 2).

A simple solution would be to scale up the total dose, such that the lung‐adjacent tumor periphery still receives sufficient dose. The resultant high dose gradient in the PTV is accepted, and even encouraged, for ablative stereotactic treatments by many groups.^9^, ^21^, ^24^ The different arguments in favor of this dose inhomogeneity are all based on the high‐dose region being concentrated in the tumor and not in healthy tissue.^9^, ^21^, ^23^ For an island‐type lung tumor, where the low‐density lung surrounds the tumor on all sides, these arguments may track well, and the dose scaling approach may be quite tenable.

In this study, we will instead concentrate on LW‐seated tumors where simply scaling up the dose may be more problematic. The close proximity of the tumor and similar‐density tissues outside the lung means that the higher‐dose region is pushed toward the lung wall and frequently into the surrounding structures. When the dose is scaled up to ensure the tumor in the lung is not underdosed, other normal tissues in this region may receive prohibitively high doses. For the tumor near the chest wall in (Fig. 1b), for example, scaling up the dose to the PTV could result in high dose to the ribs, a nearby organ at risk (OAR), potentially resulting in pain and/or fracture.

For LW‐seated tumors treated with IMRT‐based SBRT, the challenge is to use the tools currently available in treatment planning systems to both a) prevent underdosing in the lung tumor border region, and b) prevent severe overdosing of the critical OARs embedded outside the lung near or within the PTV, such as the ribs. In this work, we present a nonstandard, yet highly streamlined and practical approach to achieve these goals, and show by direct comparison that it can be superior to more standard approaches.

## II. MATERIALS AND METHODS

A database of 15 clinically treated SBRT cases with LW‐seated tumors was collected for retrospective dose computation (Table 1). As shown in Table 1, the tumors treated in these cases had a variety of sizes and locations within the lung (upper lobe, lower lobe, left lung, right lung). Treatment plans for each case were created in the iPlan treatment planning system version 4.1 (BrainLAB AG, Feldkirchen, Germany) which has both PB‐ and MC‐based dose calculation algorithms. Our group has previously reported on commissioning and initial clinical experience with this planning system.^(11)^ To create a treatment plan, each patient was first simulated on a Philips Brilliance CT Big Bore scanner (Koninklijke Philips Electronics N.V., Amsterdam, Netherlands). A four‐phase, phase‐based retrospective 4D CT scan was acquired for each patient using the Varian real‐time position monitoring (RPM) system (Varian Medical Systems, Palo Alto, CA) for respiration monitoring. Each of the four individual phase CT images was then combined to create a maximum intensity projection (MIP) image‐based CT. A free‐breathing CT was also acquired for each patient.

**Table 1:** Database of clinically planned and treated SBRT lung cases with lung wall‐seated tumors used in this study. Site describes the location within the lung as right or left lung, upper or lower lobe (RUL, LLL, etc.), and ITV volume is defined from MIP images acquired from a four‐phase retrospectively gated 4D CT.

*Patient Number*	*Site*	*ITV Volume (cc)*	*Nearby OAR*	*Dose/fx*	# *of Fxs*
1	LUL	2.75	Ribs	12	4
2	RUL	12.13	Ribs	12	4
3	RLL	73.82	Ribs	12	4
4	LUL	4.11	Ribs	12	4
5	LUL	9.47	Ribs	12	4
6	RUL	20.58	Ribs	12	4
7	RUL	12.12	Trachea	12	4
8	RLL	27.97	Ribs	12	4
9	RUL	7.58	Ribs	12	3
10	LUL	17.95	Ribs	12	3
11	RUL	4.62	Ribs	12	4
12	LLL	1.532	Ribs	12	4
13	RUL	10.305	Ribs	12	4
14	RUL	14.019	Ribs	12	4
15	LUL	20.174	Ribs	8	5

After all of the CT image sets had been exported to iPlan and registered, planning was performed on the free‐breathing CT. The MIP image was used as a guide to contour an internal target volume (ITV) surrounding the tumor. A planning target volume (PTV) was then created by expanding the ITV 3 mm isotropically. Critical structures, such as the lung and any OARs near the tumor (i.e., the ribs for tumors adjacent to the chest wall), were also contoured.

Treatment plans were created using seven coplanar beams, arranged isocentrically around the ITV. Beam angles were chosen such that they would transmit through a minimal amount of lung tissue before reaching the tumor while avoiding collisions with the table and patient. PB‐based IMRT optimization was then performed with the goal of delivering the prescription dose to 95% of the PTV. All but three of the plans were prescribed 4 fractions (fx) of 12 Gy (48 Gy total). Two patients received only 3 fx of 12 Gy (36 Gy total) and one patient was prescribed 5 fx of 8 Gy (40 Gy total), both due to previous treatment to the same area of the lung. Treatment constraints for the OARs followed the guidelines for lung SBRT from RTOG Protocol 0236.^(1)^

Following PB‐based optimization, the final dose distribution was recalculated with MC. To achieve sufficient PTV coverage following MC dose calculation, the dose is renormalized such that 95% of the PTV receives the prescription dose. This scaling‐based approach (the “standard method”) is the typical methodology used in our clinic. Treatment plans for all cases in the database were created using two alternate techniques and then compared to the standard technique.

The first of these, “iterative reoptimization,” follows the standard technique up to the MC dose calculation. An isodose line is first selected as the boundary between the high‐dose and low‐dose region of the PTV. The volume of the PTV receiving a dose less than this isodose, excluding any of the PTV region extending outside the lung, is contoured and designated as the low‐dose region of the PTV (such as the low‐dose region in (Fig. 2b), for example). The plan is then reoptimized with the contoured low‐dose region designated as a “boost structure” and assigned a treatment planning goal to receive an additional dose above the prescription dose. After reoptimization, the final dose is calculated using MC, and the plan is then renormalized to deliver the prescription dose to 95% of the PTV. Essentially, the iterative reoptimization technique determines the underdosed region of the tumor during the first optimization. The second optimization then increases the dose calculated by the PB algorithm to this underdosed region. With a single additional optimization step, this mitigates the PTV underdosing, compared to use of a scaling technique alone, resulting in less enhancing of the hot spot within the nearby OAR to achieve proper PTV coverage.

The iterative reoptimization technique requires selection of a dose goal for the boost structure and a proper isodose line to designate the boundary between the high‐dose and low‐dose region of the PTV following the initial optimization. For consistency in this study, a single set of values for both of these was used. These were determined from a randomly selected subset of cases from the patient database. Across this subset, it was determined that, after the initial optimization, the periphery of the PTVs received, on average, 75% of the prescription dose. Thus a boost dose of 25% of the prescription dose was chosen for use with the entire database. It was also noted that in all of the subset cases, the 103% isodose line (within our clinical convention of prescribing to the 95% isodose line) provided a reasonable boundary for the underdosed region following the initial optimization and was thus used with the entire database of cases.

The iterative optimization method was compared against a multiple noncoplanar beams approach which is more widely used to achieve greater plan quality for LW‐seated tumors. Before optimization, in addition to the seven coplanar beams, five noncoplanar beams were placed using the lung contours as a guide to select beam angles that would penetrate a minimal amount of lung tissue before reaching the tumor while avoiding collision with the patient or table. As with the other techniques, following optimization and final dose calculation with MC, the plan was renormalized until 95% of the PTV received the prescription dose.

The differences in plan quality between the standard method and the other two techniques for the PTV were assessed in each plan by comparing the conformity index (CI), defined in iPlan as:^(25)^

(1) CI=(PIV/TV)/(TC)

where *PIV* is the volume enclosed by the prescription isodose line, *TV* is the tumor volume (in this case the PTV volume), and *TC* is the target coverage, the percentage of the target covered by the prescription isodose. A measure of uniformity, $U_{\text{PTV}}$, given by:

(2) $$U_{PTV}=d_{\max PTV}-d_{\min,PTV}$$

where $d_{\text{max},PTV}$, $d_{\text{min},PTV}$ are the maximum and minimum doses received by the PTV, respectively, was also analyzed. The measured CI and $U_{\text{PTV}}$ values for each of the three planning techniques are shown in Table 2 for all cases in the database.

**Table 2:** Values of the conformity index (CI) and the uniformity ($U_{\text{PTV}}$) determined for each case in the database when planned using the standard method, iterative reoptimization, and using noncoplanar beams. CI is unitless while $U_{\text{PTV}}$ is in units of Gy. Note that lower values of both CI and $U_{\text{PTV}}$ are considered superior.

*Patient*	*CI*			$U_{\mathrm{PTV}}$		
	*Standard Method*	*Iterative Reoptimization*	*Noncoplanar Beams*	*Standard Method*	*Iterative Reoptimization*	*Noncoplanar Beams*
1	1.59	1.19	1.41	25.04	15.39	28.95
2	1.39	1.25	1.30	13.94	13.28	16.03
3	1.12	1.24	1.08	24.53	25.7	14.58
4	1.31	1.13	1.53	17.33	15.81	17.44
5	1.38	1.36	1.34	19.85	13.08	21.69
6	1.40	1.23	1.24	18.94	16.14	17.69
7	1.16	1.52	1.24	21.13	27.8	23.07
8	1.21	1.35	1.17	10.9	10.63	15.72
9	1.44	1.34	1.45	14.94	10.18	15.34
10	1.61	1.22	1.55	20.24	17.31	19.89
11	1.63	1.31	1.58	29.24	14.73	28.2
12	2.24	1.81	2.47	28.33	20.12	27.23
13	1.26	1.14	1.21	15.04	12.96	15.55
14	1.37	1.13	1.47	19.24	10.37	21.45
15	1.48	1.47	1.19	13.89	14.73	14.38

In each case, for the nearby OAR of interest, the dose to 1 cc of this structure was determined for all three methods (Table 3). This metric was chosen as the nearby OARs to the LW‐seated tumor are especially sensitive to the maximum dose (or dose to a small volume) in the treatment plan. For example, the ribs are a common nearby OAR and do not demonstrate a volume effect, but rather a threshold dose effect; if too high a dose is applied, then pain and fracture can result.^26^, ^27^ It is also important to have a metric to assess the low‐dose spillage, as this could change depending on the method used. For stereotactic procedures, this is frequently done using a gradient index (GI), such as that developed by Paddick and Lippitz.^(28)^ However, for lung SBRT, the low‐dose spillage is likely to have the greatest impact on complications in the lung. Evidence in the recent literature suggests that the volume of lung receiving at least 5 Gy (V5) is a good metric to assess the impact of the low‐dose bath on the lung,^29^, ^31^ and will be the metric used here. The measured V5 values for all three planning techniques are shown in Table 3.

**Table 3:** Values of the dose to 1 cc of the nearby OAR (D1cc) and the volume of lung receiving 5 Gy (V5) determined for each case in the database when planned using the standard method, iterative reoptimization, and using noncoplanar beams. The nearby OAR for each case is listed in D1cc is given as a percentage of the prescription dose, while V5 is in terms of percentage of the total lung volume.

*Patient*	*D1cc for Nearby OAR*			*Lung V5*		
	*Standard Method*	*Iterative Reoptimization*	*Noncoplanar Beams*	*Standard Method*	*Iterative Reoptimization*	*Noncoplanar Beams*
1	115.9%	99.9%	108.0%	8.4%	8.2%	9.0%
2	106.8%	96.1%	96.0%	14.8%	15.3%	16.9%
3	109.1%	110.0%	107.0%	17.8%	19.4%	16.9%
4	106.9%	96.3%	107.0%	7.9%	7.9%	10.0%
5	107.9%	97.5%	93.0%	12.0%	12.4%	9.8%
6	109.0%	103.7%	109.0%	19.6%	19.0%	19.4%
7	114.9%	102.6%	114.9%	14.7%	15.0%	12.6%
8	103.1%	98.8%	104.4%	20.6%	21.1%	16.0%
9	100.0%	88.8%	88.1%	6.1%	6.2%	5.4%
10	109.1%	90.1%	111.8%	35.8%	33.9%	33.6%
11	102.9%	86.3%	93.1%	7.0%	7.1%	6.7%
12	104.9%	91.0%	106.0%	3.9%	3.5%	3.1%
13	108.0%	96.2%	102.0%	15.6%	15.3%	13.6%
14	114.9%	98.6%	111.9%	19.1%	17.9%	17.0%
15	103.1%	99.7%	103.0%	6.8%	6.9%	7.9%

For each metric, the magnitude and frequency of improvement versus the standard technique across all cases was tabulated. To assess the overall ability of a given technique to improve upon the standard method, one‐tailed paired Student's t‐tests were performed on each metric for all cases.

## III. RESULTS

**Figure 3:**
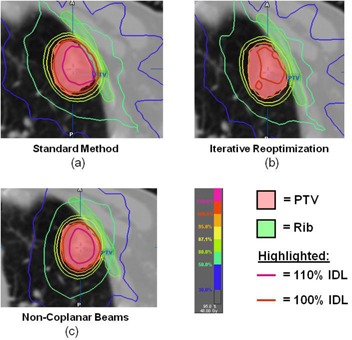
Example of a MC‐calculated treatment plan for a LW‐seated tumor using (a) standard method, (b) iterative reoptimization, and (c) noncoplanar beam technique with 110% and 100% isodose lines (IDLs) highlighted. In the standard technique (panel a), note that the hot spot is pulled past the tumor center and into the nearby ribs, while a high dose gradient exists on the lung‐embedded side of the tumor. Using iterative reoptimization (panel b), the hot spot has been reduced (note that the 110% IDL line is not longer there) and moved away from the ribs, and the large gradients across the tumor are absent. When using noncoplanar beams (panel c), the hot spot has been moved into the tumor center, reducing the high dose to the ribs and pushing the high dose gradients to the tumor periphery.

An example of the typical results seen using all three planning methods is shown in Fig. 3. A typical LW‐seated tumor planned using the standard method, shown in (Fig. 3a), has a hot spot that is not centered on the tumor, but is rather pulled over into the chest wall and the OAR (ribs) therein. There is also a very large dose gradient across the lung‐embedded side of the tumor.

**Figure 4:**
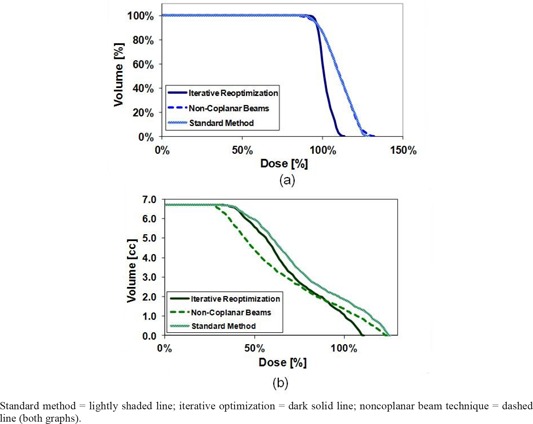
Dose‐volume histograms (DVHs) for the (a) PTV and (b) rib structure seen for the plans in Fig. 3.

The dose gradient across the PTV and the high‐dose intrusion into the ribs can be seen in the dose‐volume histograms for these structures, seen in the light, shaded lines in Fig. 4.

The iterative reoptimization technique addresses many of these issues, as seen in (Fig. 3b). In the PTV, the hot spot is reduced considerably (note in (Fig. 3b) the disappearance of the 110% isodose line (IDL) compared to (Fig. 3a), and moves centrally into the tumor, away from the ribs. In addition, the sharp dose gradients across the PTV have been greatly reduced in this plan. The DVH for this plan, seen as the dark solid line in Fig. 4, has a much sharper falloff for the PTV than compared to the standard method. Moreover, the rib DVH demonstrates a faster falloff in dose, resulting in a reduction of the maximum dose delivered. Finally, it should be noted that planning using the iterative reoptimization technique took, on average, around 1.5 times that for creating a standard plan on the same patient, largely due to the additional time needed for contouring (data not shown). For example, if standard planning required 1 hour for a given patient, then it could be expected that planning using iterative reoptimization on that patient may take around 1.5 hours.

(Fig. 3c) shows the noncoplanar beam plan. Note that the hot spot seen for the standard method in (Fig 3a) has not been reduced. Instead, it has been moved interiorly towards the center of the tumor and away from the ribs. Large dose gradients still exist within the plan; however, as the hot spot is pulled centrally into the tumor, while the large gradients are pushed to the PTV edge. The DVHs for the noncoplanar plan — the dashed line in Fig 4 — lies for the PTV almost completely on top of the standard method DVH. While there are differences in the plans, as seen in (Figs. 3a) vs. 3(b), the similarity between the DVHs illustrates the hot spot remaining within the PTV. (Fig 4b), the rib DVH, shows a reduction of the dose to 1 cc of the ribs for noncoplanar beams compared to the standard method. Thus, these DVHs reflect that the retained hot spot has been moved from near the ribs toward the center of the tumor. Planning took a similar, although typically longer, amount of time as that for the standard technique, as some additional time was needed to find a useful placement of the nonplanar fields.

Standard method=lightly shaded line; iterative optimization=dark solid line; noncoplanar beam technique=dashed line (both graphs).

The quantitative results for the PTV are shown in Table 4. In comparison to the standard method, the iterative reoptimization technique offered improvement in the CI and the $U_{\text{PTV}}$ for 80% of all cases, with an average improvement of 13% ±2% and 24% ±5%, respectively. Across the entire database of cases (i.e., including those which showed improvement compared to the standard technique and those which did not), the one‐tailed t‐test p‐values showed that the improvements were statistically significant at the p=0.05 level, with p < 0.03 for CI and p<0.01 for $U_{\text{PTV}}$. Comparison of the noncoplanar beams versus the standard method showed improvement in 67% of cases for CI and 33% for $U_{\text{PTV}}$, while the average improvements for these cases were found to be 7% ±2% for CI and 11% ±7% for UPTV. These values are all less than what was found for iterative reoptimization. Furthermore, the noncoplanar beam results did not show differences which were statistically significant compared to the standard method, with p=0.27 for CI and p=0.36 for UPTV.

**Table 4:** Percent increase in PTV metrics vs. standard technique: improvement for the PTV across all plans in the database comparing the iterative optimization technique and the use of noncoplanar beams versus the standard method. Both the conformity index (CI) and the uniformity ($U_{\text{PTV}}$) are shown with the values tabulated from Average standard error and range are the % increase only, while the p‐values consider if the improvements are significant over all cases in the database.

	*CI*				$U_{PTV}$			
	*% of Cases Showing Improvement*	Average ± Standard Error	*Range*	*p‐value*	*% of Cases Showing Improvement*	Average ± Standard Error	*Range*	*p‐value*
Iterative Reoptimization	80.0%	13.4%±2.3%	[1.0%‐25.0%]	0.02	80.0%	24.0%±4.7%	[2.5%‐49.6%]	<0.01
Noncoplanar Beams	66.7%	6.9%±1.7%	[2.6%‐19.6%]	0.27	33.3%	11.3%±7.4%	[1.7%‐40.6%]	0.36

Similar results were found for the nearby OARs, as shown in Table 5. The iterative reoptimization technique showed improvement (reduction) of the dose to 1 cc of the OAR versus the standard method for 93% of all cases (average improvement 11% ±1%), compared to only 67% of cases for the use of noncoplanar beams (average improvement 6% ±2%). As before, across all plans in the database, these differences showed statistically significant improvement for the iterative reoptimization technique (p < 0.0001) although unlike in the PTV, the improvements were also statistically significant for the use of noncoplanar beams (p < 0.01).

**Table 5:** Reduction in the dose to 1 cc of the nearby OAR (ΔD1cc) and reduction in the lung volume receiving 5 Gy of dose (ΔV5) across all plans in the database comparing the iterative optimization technique and the use of noncoplanar beams versus the standard method. The nearby OARs are as noted in each case in Average, standard error, and range are the % increase only, while the p‐values consider if the improvements (decreases) are significant over all cases in the database.

	*Nearby OAR* ‐ Δ *D1cc*				*Lung* ‐ Δ*V5*			
	*% of Cases Showing Improvement*	Average ± Standard Error	*Range*	*p‐value*	*% of Cases Showing Improvement*	Average ± Standard Error	*Range*	*p‐value*
Iterative Reoptimization	93.3%	10.7%±1.1%	[3.3%‐17.4%]	<0.0001	46.7%	0.6%±0.3%	[0.0%‐1.8%]	0.43
Noncoplanar Beams	66.7%	6.2%±1.6%	[0.1%‐13.8%]	<0.01	73.3%	1.6%±0.4%	[0.2%‐4.6%]	0.053

Results for the lung V5 values, also shown in Table 5, show that the choice of treatment planning algorithm does not greatly affect the low‐dose spread. The use of noncoplanar beams did a slightly better job of reducing the low‐dose spread versus the standard method compared to the use of iterative reoptimization. Noncoplanar beams showed improvement compared to the standard method in 73% of all cases (average improvement 2% ±0%), while the V5 improved in only 47% of all cases using iterative optimization (average improvement 1% ±0%). However, both of these average improvements are minimal, and the results from neither technique showed statistical significance compared to the standard method at the p=0.05 level (Table 5).

The iterative reoptimization technique did not improve upon the standard method in all cases. In terms of PTV coverage, in two cases out of fifteen (Patients 3 and 7 in Table 1), both the CI increased (by 11% and 31%, respectively) and the $U_{\text{PTV}}$ decreased (by 5% and 32%, respectively), failing to show improvement in CI and $U_{\text{PTV}}$. In two cases, one of the PTV metrics did not show improvement — the CI increased in one case (Patient 8) by 11%, and the $U_{\text{PTV}}$ decreased by 6% in the other (Patient 15). For Patient 3, where both the CI and $U_{\text{PTV}}$ were worse for iterative reoptimization, the dose to 1 cc of the OAR increased by 1% compared to the standard method, as well.

**Figure 5:**
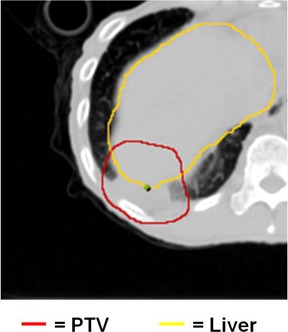
Image from a case in which the iterative optimization method did not improve the analyzed statistics compared to the standard method. Note the proximity of the PTV to the skin and the large amounts of nonlung (unit‐density) tissue within the PTV.

Ultimately, the cases which did not show improvement had one or more of the following features: a) the PTV contained a large amount of nonlung (near unit density) tissue, b) the PTV is very large, and/or c) nonlung structures near the PTV were filled with very low‐density material (examples: the skin or an air‐filled trachea). An example of multiple issues is seen with Patient 3, an image from whom is shown in Fig. 5. Here, the ITV (and hence, PTV) for this case was large (over 2.6 times larger than the next largest ITV in the database; see Table 1), close to the skin, and contained a large amount of nonlung tissue, which more closely resembled “unit density” tissue. Patient 7 showed the largest negative changes in CI and $U_{\text{PTV}}$. In this case, the nearby OAR was an air‐filled structure (trachea), the loss of CPE in which might have altered the dose distribution therein. This loss of CPE in the OAR was not accounted for in the iterative optimization technique as used here.

It is also important to note that there were several cases where the noncoplanar beams did not show improvement compared to the standard method (maximum increase in CI of 18%, decrease in $U_{\text{PTV}}$ of 44%, and increase in the dose to 1 cc of OAR of 3% all occurred in different cases), although unlike with the iterative reoptimization cases, there did not seem to be an easily discernible pattern for which cases this would occur.

## IV. DISCUSSION

For PB‐algorithm, IMRT‐based, lung SBRT treatment planning of LW‐seated tumors with the final dose calculation done using MC, the iterative optimization technique was a practical, streamlined approach which proved to be superior overall to both the standard method and the use of noncoplanar beams, based on the metrics evaluated in this study. The one‐tailed paired t‐tests showed that it offered a statistically significant improvement in all of the metrics analyzed across the entire database of cases, compared to the standard method (except the V5, which showed no statistically significant differences for any of the planning techniques analyzed). Furthermore, in comparison to the standard technique and the noncoplanar beams technique, iterative reoptimization not only improved all metrics for a larger percentage of cases, but also resulted in higher average percentage improvements. This may be due in part to the small range of noncoplanar beam angles available for some SBRT lung cases due to couch and patient collision issues (our clinic, for example, restricts couch angles to ±10° from zero for anterior treatments). This could limit the potential improvements achievable through use of noncoplanar beams. Regardless, the metrics utilized assessed both PTV coverage and hot spots in the nearby OARs. Improvements in the metrics seen for the iterative reoptimization technique compared to the other methods imply it is better able to achieve the LW‐seated tumor planning goals of providing sufficient target coverage and reducing dose to the nearby OAR. Furthermore, although planning using the iterative reoptimization technique took longer, as described above, the additional time needed was still quite practical and was not, from our experience, detrimental to the clinical workflow.

The use of noncoplanar beams showed improvement based on the metrics evaluated here compared to the standard method for a predominant number of cases in terms of both improving CI and reducing maximum dose to the nearby OAR. Noncoplanar beams did not frequently show improvement in the UPTV parameter. This relates to the fact that the use of noncoplanar beams mitigated the high dose to the OAR by shifting the hot spot away from the OAR and more centrally into the PTV instead of reducing it. As discussed above, such dose distributions, as they would be in island‐style tumors, is not regarded as inherently problematic. It could be thus be argued that $U_{\text{PTV}}$ is an improper metric to assess the use of noncoplanar beams for LW‐seated tumors. However, with noncoplanar beams, the high‐dose region is still close to the sensitive OAR. Movement and/or errors in treatment setup could shift the hot spot back into the nearby critical structure. This is not as problematic when using iterative reoptimization as the hot spot is reduced while maintaining proper target coverage. This is in addition to the fact that iterative reoptimization showed superior improvements in the metrics compared to the use of noncoplanar beams.

The use of noncoplanar beams was superior the iterative reoptimization technique in terms of the V5 values, a measure of the low‐dose spread, showing improvement compared to the standard method in more cases (73% vs. 47%). However, the changes were small enough (average improvement of 2% for noncoplanar beams vs. 1% for iterative reoptimization) that the changes in these values would not have been a determining factor as to which plan to use in our clinic. This minimal change in the low‐dose spread between the different optimization techniques is reinforced by the lack of statistical significance in the differences seen in the V5 values across all methods analyzed.

As noted earlier, the iterative reoptimization technique did not show improvement compared to the standard method in all cases. Although we were able to qualitatively recognize consistent features of plans that did not show improvement, (large PTVs, PTVs with a high amount of nonlung, unit‐density tissue, and PTVs near nonunit density containing/adjacent structures such as the skin or trachea), a prospective algorithm for determining which cases iterative reoptimization would be useful has not yet been determined. The limited number of cases with diminished metrics from iterative reoptimization compared to the standard technique makes creation of such an algorithm difficult; this speaks to the efficacy of iterative reoptimization, but also implies that a broader analysis of more cases may be useful for this endpoint. It is also important to note, however, that in none of the cases did all of the metrics analyzed fail to show improvement. This complication could make it far more challenging to create a prospective algorithm to determine which cases the iterative reoptimization technique is not beneficial.

A related issue is that some of the limitations of the iterative reoptimization technique may be due to the fact that certain parameters used in iterative reoptimization were defined absolutely across all plans — the isodose level used to denote the underdosed region of the tumor, and the percentage dose used to boost that region. As noted earlier, the values for these were based on a subset of cases in the database and were applied universally to limit the variability the process of deciding such values on a case‐by‐case basis could induce on the results. However, selection of individual case‐based parameters for the iterative reoptimization process could potentially allow for improvements in the evaluated metrics in cases where they were not currently seen, as well as yield greater improvements in the cases where iterative optimization had previously shown to be beneficial.

There are other caveats to note regarding this study. The metrics employed in this study are limited in their ability to completely describe the quality of a plan. For example, it could be argued that, in some cases, the use of noncoplanar beams results in better coverage on the superior side of the PTV, which was not discussed here. However, we have attempted to select complementary metrics to give a sense of the impact that different planning methods can have regarding important issues which can impact LW‐seated SBRT lung treatments. A CI variant was chosen, for example, which includes the target coverage by the prescription isodose line, an aspect which speaks directly to the issue of ensuring that the cold edges of the lung‐adjacent tumor are properly covered. Treatment planners, however, will use more tools in combination to assess plan quality: metrics, isodose and volume contours, and DVHs. In Figs. 3 and 4, examples were given of these latter two, which were not atypical results for this study, but each plan would have an independent clinical assessment before delivery.

This study has also been limited to IMRT approaches to lung SBRT planning. Due to the motion of some lung tumors and issues such as the interplay effect with the leaf motion of the multileaf collimator (MLC), it has been noted that IMRT for hypofractionated lung tumors could be problematic, an issue which is minimized with a 3D approach.^32^, ^34^ The iterative reoptimization technique could increase the modulation factor, which could have an impact on the interplay effect, although the degree of this impact is unknown. Ultimately, the resolution of such issues is beyond the scope of this study. However, as SBRT is heavily intertwined with image guidance, and as motion evaluation and management techniques continue to improve, IMRT may become more prevalent for lung SBRT, especially for tumors with limited excursion or those patients for whom tumor motion restriction techniques are applied during treatment (gated treatments, breath holds). Furthermore, an iterative approach similar to the IMRT‐based one used here could be applicable to 3D plans with Monte Carlobased dose calculation.

These last few points provide the impetus for the future directions of this project. The parameter selection for the iterative reoptimization method needs to investigated and optimized such that both the isodose line used for contouring the cold, underdosed region of the tumor and the dose goal given to the boost structure can be selected based on aspects of an individual case. Development of a prospective method for determining which plans would benefit from iterative reoptimization and would also be useful. Given that both iterative reoptimization and using noncoplanar beams offered improvements over the standard method in a majority of cases, another avenue is to explore whether they would have a greater impact if used in combination. Finally, given the prevalence of 3D‐based approaches to lung planning, it would be interesting to analyze the impact of iterative reoptimization IMRT‐based planning compared to 3D approaches in terms of both clinical throughput and plan quality, and/or to investigate an iterative approach with 3D‐based planning.

## V. CONCLUSIONS

Treatment planning systems employing MC‐based dose calculation algorithms are becoming more readily available in commercial systems. This provides the opportunity to more accurately assess the dose distribution in plans of sites where other algorithms have difficulty, especially a situation with interfaces between low‐density and high‐density materials, such as in the lung. However, the reliance on PB algorithms for IMRT optimization can render final plans suboptimal. For lung SBRT treatments with LW‐seated tumors, this can be especially problematic, with hot spots pulled out of the tumor and into nearby OARs, such as the ribs. The iterative reoptimization technique developed here presents a straightforward, practical approach which may be able to create superior treatment plans for a majority of these cases. As the technologies which would necessitate the use of such a methodology become more prevalent, iterative reoptimization could become an important tool to creating high‐quality, MC‐calculated, IMRT‐based plans for LW‐seated tumors treated with SBRT.

## ACKNOWLEDGMENTS

This work supported in part by NIH/NCI Grant No. 106770.
